# COGNITIVE INTRAINDIVIDUAL VARIABILITY TO MEASURE INTERVENTION EFFECTIVENESS: BALTIMORE EXPERIENCE CORPS TRIAL

**DOI:** 10.1093/geroni/igz038.2600

**Published:** 2019-11-08

**Authors:** Allison A Bielak, Christopher R Brydges, Michelle C Carlson, Ryan M Andrews, George W Rebok

**Affiliations:** 1 Colorado State University, Fort Collins, Colorado, United States; 2 Johns Hopkins Bloomberg School of Public Health, Baltimore, Maryland, United States; 3 Leibniz Institute for Prevention Research and Epidemiology – BIPS, Bremen, Germany

## Abstract

Studies investigating the effectiveness of intervention programs on cognitive ability in older adults are equivocal; however, these studies generally focus on traditional measures of cognition, and therefore may miss some improvements by not utilizing alternate measures. We evaluate the potential for intraindividual variability in cognitive speed (IIV), a demonstrated sensitive indicator of cognitive functioning, to be used as an index of cognitive plasticity from an intervention. The current study evaluated whether older adults in a school volunteering program showed a reduction in IIV, compared to a low-activity control group over two years of exposure. Non-demented aging older adults (n = 336) participated in the Baltimore Experience Corps Trial, an evaluation of a volunteering program conducted at elementary schools designed to increase older adults’ physical, cognitive, and social engagement. Participants completed a cognitive battery that included a computerized Stroop task at baseline and after 12 and 24 months. Participants who complied at the 80th percentile or above showed a significant reduction in IIV at 24 months, with an additional trend of improved IIV with increased compliance to the treatment protocol, both at 12 months, and at 24 months. Men specifically also showed significant dose-dependent improvements after 12 months. The Experience Corps program resulted in an improvement in cognitive performance as measured by IIV. Analyzing previously collected data with non-traditional measures of cognition, such as IIV, may be a potentially fruitful and cost-effective method for understanding how interventions impact cognition in aging populations.

